# Clinical Lecture upon Spinal Curve

**Published:** 1877-08

**Authors:** J. F. Miner


BUFFALO

### Medical and Surgical Journal.

VOL. XVII. AUGUST, 1877. No. 1.

### Original Communications.

———:o:———

ART. I.—*Clinical Lecture upon Spinal Curve.* By PROF. J. F. MINER, M. D. Reported by CHAS. A. WALL, B. S., Member of the Class.

*Gentlemen:*—The Spinal Column, in the adult, consists of twenty-four single bones, (*vertebræ*) united in a continuous column by twenty-three inter-vertebral fibro-cartilages and held together and sustained by a strong ligamentous apparatus. Extending through the centre of this column is the spinal cord; (*medulla spinalis*) with the delicacy of whose structure, and importance of whose function you are all sufficiently familiar. The form or curves, together with the intervening fibro-cartilages, endow the spine with a high degree of elasticity and flexibility, which is regulated by the shape of the vertebral bones, and by the ligamentous apparatus by which the bones are strongly united. It has been believed that the curves not only add to its flexibility, but also to its strength, but such a view is opposed to all mechanical law. The curves render the spine more secure from violence and less liable to suffer from a shock, but cannot be shown to contribute to its natural strength.

The spine in infancy and childhood differs widely from that of adult life. The ossification in the embro of the spine appears later than in almost any other part of the skeleton; its anatomical perfection requires more time, and extends even beyond the period of puberty. During all this period, some parts of the vertebræ are connected with the bodies by cartilages, and are consequently more easily separated than when the ossification is complete. This is to be borne in mind, in connection with the fact, that curvatures of the spine may occur in adult life, but are mainly observed in infancy and childhood, and your attention is called to this subject on account of the frequency of its occurrence, and the importance of early recognition and proper treatment. This disease has been studied anew, within the past few years, and views formerly considered as well founded and settled are now believed to be unsustained by clinical observation.

*Lateral Curvature* of the spine is a much less serious and a very different disease from that produced by caries of the vertebræ, and known as angular or posterior curvature of the spine. Lateral curvature is never so abrupt and consequently its effects upon the spinal cord are less serious; it is rarely, if ever, attended by caries of the bone or ulceration of the inter-vertebral cartilages, and on this account is quite unlike angular curvature. Its common causes appear ofter quite unlike angular curvature. Angular curvature depends upon organic change in the vertebræ or the inter-vertebral cartilages, while the lateral, or sideways curves, appear generally as caused by irregular muscular contraction, acting upon weakened, irritated, or otherwise diseased bones, cartilages and ligaments, drawing them out of their natural position and inducing deformity.

The *causes* which may produce this irregular muscular action are various and require brief notice. Hypertrophy of the muscles may be induced simply by excessive use, in following any avocation requiring constant use of one arm, or of the muscles of one side. In this way the spine may be made to deviate laterally, since it is a law that muscles grow from use and become atrophied from rest. The muscles of one side becoming more fully developed and powerful, overcome their fellows of the opposite side and produce marked curvature, the convexity of which points to the hypertrophied limb. Similar effects are produced if the muscles on one side of the spine become atrophied and loose their power, while on the other side the muscles retain their natural vigor and strength. This is sometimes observed when muscles become disabled from inflammation as in rheumatism, or by paralysis, as in severe contusion, and in failure in nervous influence, or by spasmodic contractions as in wry neck, which, whenever it occurs in severe form, is always accompanied by curvature of the cervical portion of the spine, often in a distressing degree.

Debility of the muscles is a frequent cause of curvature, probably the most frequent of all. Thus local debility is often caused by *fatigue* of the muscles of the back from protracted maintenance of the erect posture and without support for the spinal column, as is common in factories, and some schools. The muscles soon become wearied and give way to the stronger ones of the opposite side, or to the less wearied antagonists.

*Obliquity of the pelvis*, is always followed, if long continued, by lateral deviation in the spine, especially is the deformity to be seen in the lumber region. This fact has suggested a method of cure based upon the fact that obliquity producing deviation; it might be instituted as a means of cure. No doubt some success might attend in selected cases, the adoption of the plan, viz.: seating the person upon an inclined plane, the inclination being suited and regulated to meet the requirements of each case. An altered state of the chest, as occurs from pleurisy greatly diminishing the capacity of the the thorax on the affected, and increasing it on the opposite side, often produces lateral curvature. The disease of early life known as *Rachitis*, where the bones are greatly deficient in the earthy salts, with corresponding increase in the animal constituents, thus making the bones incapable of withstanding the action of the various muscles or supporting the weight of the body, is often attended by curvature in the spine, in common with almost all the other bones of the skeleton.

Defective development or malformation of the vertebræ, by conjenital defect will cause lateral deviation in the spine, but it will be observed that not one of these conditions has a tendency to end in caries, or ulceration of the bone.

Thus far in enumerating the causes of lateral curvature, I am sustained by all authority, but I cannot withhold expression of an opinion, that in some instances at least, the primary seat of the disease is in the bones or inter vertebral fibro-cartilages, and that reflex muscular contraction is induced by it, thus drawing upon one side disproportionately and causing lateral curve. The form of the vertebræ is such, that lateral curve cannot take place so abruptly as posteior curve. The spine rarely curves laterally except by long arches, and this is fully accounted for in the shape of the bones without going back to the causes of the disease, and its pathological conditions. If lateral curve was produced by caries of bone or by ulceration of cartilage, the angles would not be sharp as in posterior curvature, for obvious reasons, and the same anatomical conditions prevent the lateral curve from producing serious ill effects. In the common cases of lateral curvature, the deformity begins in the upper dorsal vertebræ which are more frequently drawn to the right side. One curve being thus formed, nature, always ready in instituting measures of restoration, commences by forming compensating curves, and we soon have two others to counter-balance the first and maintain the equilibrium, one in the cervical and one in the lumbar region, their developement occuring simultaneously, and of course in a direction opposite to the primary curve. The vertebral column is diminished in length in proportion to the curves; the chest is greatly altered in figure the ribs being flattened, elongated and twisted, and the sternum and costal cartilages being tilted forwards and depressed upon the pelvis. The scapula is elevated and rendered prominent on one side and depressed on the other, curve in the cervical region gives obliquity to the head, and in the lumbar region, to the pelvis.

This is however denied by some who declare that scoliosis (lateral curvature) is never tracable to traumatic injury such as is liable to lead to structural change in the bones or cartilages.

The *prognosis* in recent cases of lateral curvature, is generally in those of comparatively healthy constitution. Proper management will then usually effect complete restoration although it must be early instituted and perseveringly followed. When the affection depends upon paralysis of the spinal muscles, organic disease of the vertebræ or cartilages and ligaments, or serious lesion of the pelvis, hip or leg, complete cure will be impracticable. Some cases of long standing may be regarded as incapable of perfect cure.

*Treatment.* In speaking of the various causes of lateral curvature, I have indicated that all cases are not alike, and not amenable to the same treatment; hence before any measures are instituted for its relief, the most careful inquiry should be made in reference to its exciting cause. So long as the cause under whose influence the disease has been developed is allowed to continue in operation, it will be impossible to make progress toward cure. The discontinuance of any employment which has been acting as a cause, will often speedily allow of restoration, by enabling the muscles of the two sides to regain their equilibrium upon the loss of which the trouble depends. Thus if curvature is caused by rest of one arm and constant use of the other, reverse this action; if from position at school or in factory from viscious habits of standing or sitting, rectify and change these conditions. In cases of debility and anæmia, tonics are indicated the whole list being proper, and suitably joined with other hygenic conditions are often very useful and proper, never however forgetting that the best and most active tonics in the world, are *food, exercise in the open air, warm clothing and general good care.* Iron and Bark are properly tonics, but of little benefit unless associated with the other conditions. Cod liver oil, stimulants and indeed a long list of adjuvants might be added, —will be added to too great extent in protracted cases.

To all this, one other remedy, more important in its direct influence than all others must be added, *Mechanical Support* is proper, and indispensable in nearly all cases, and your attention must be directed to it, and you must be fully impressed with the idea that without it the treatment of spinal curvature is deficient. I have already indicated my own personal opinions that the cause, in some cases at least, oftener than generally believed, is the same as in the angular curvature, that it arises from injury, and inflammation or other change in bone or cartilage which stimulates reflex muscular action, and I may now add, that whatever the cause, *all* cases may be benefited by support. They may at least be preserved from greater deformity, relieved from pain, supported and sustained. *Mechanical Support* then is to be added to any and all other measures of treatment.

How this is applied, I will attempt to show you, and to speak a little more in detail, since my chief object is to make you practically acquainted with the proper modes of treating spinal curvature.

Much ingenuity has been exhibited in the manufacture of the various spinal apparatus for support, both in lateral and angular curvature. There is a vast amount of machinery for this purpose before the profession, all of which is more or less adapted to the object in view. Some of it is very elaborate, expensive and cumbersome; and some too flimsy and cheap to be useful. As a general rule, the lightest, most comfortable, and most simple, is the best, but each inventor or manufacturer has conbined or embodied some advantages not possessed by all others. That instrument which offers the best support, restores deformity in the greatest degree and is the least uncomfortable, is certainly to be preferred. Different cases may require different instruments. But I was proposing to consider at this time only

POSTERIOR CURVATURE OF THE SPINE.

The authors of the seventeenth century promulgated the idea that posterior curve of the spine was due to caries of the vertebral bones. This idea was extended by later surgeons, and the view that the caries was due to tubercular disease was generally entertained; and with many surgeons and authors, it prevails at the present time. Two or three modes of tubercular invasion are described,—either there is a central deposit in the vertebral bone which extends gradually and destroys the structure of the vertebral body until it is incapable of bearing the weight of the trunk, or the tubercular material is is deposited in front or by the side of the bodies of the vertebræ, and gradually excavates them until they break down, and the spine bends either posteriorly or laterally, as the case may be. To this, a third has been added, namely, a general infiltration of tubercular substance, throughout the entire cancellated structure of bone, with subsequent absorption, or formation of sequestrum.

With few exceptions, these views have been adopted by the profession. Dr. Pott, who early described the disease and after whom it was called "Pott's disease," regarded it as caries of the vertebral bones. Subsequent authors added that the caries was due to tuberculosis; thus explained, it has almost uniformly been understood.

It will appear strange that anything almost universally conceded by the whole body of the profession should be doubted, especially when the most positive assertions have been made upon this subject by surgeons of the highest respectability.

Upon a careful consideration of the facts which clinical experience constantly furnish, it seems well to again review the ground, though natural, that we approach the subject with some embarrassment. False views in pathology lead to fearful errors in practice, while a distinct and rational view of the causes of spinal curvature, will be followed by corresponding correctness in our means of cure.

Recently tubercular origin in the caries of the spine has been denied, and the evidences aduced seem to my mind conclusive that it was a mistake. Virchow asserts that he has never seen the so-called tubercular cell, and that in the matter hitherto pronounced tubercular, he had failed to observe anything unlike the results of inflammation. Experiments show that pus undergoes transformations which render it not unlike tubercular matter, semi-fluid at first, then at a later period, of cheesy consistance, then hard and finally semi-translucent. This change being due to absorption of its fluid portions. Rokistansey and others even state that pus may undergo the process of calcariazation. In this respect then, tubercular matter presents no marked difference from pus. Pathologists declare, that in numerous instances of so-called tubercular deposites in bones, the microscope revealed fat and other granules, pus corpuscles and bone detritus. In some specimens so large corpuscles have been found that it was impossible to determine whether they were tubercles or pus; these large corpuscles being perhaps the parental pus cells, now much better understood than when these observations were first made. In doubting the existance of tubercular disease of the spine, we do not dispute the caries, but only deny the tubercular cause of caries.

The greatest importance, in my opinion, may be given to this other and very commnnly observed fact, viz. Tubercular disease is one of almost uniform progress to a fatal result. We do, it is true, observe arrested tuberculosis, but in the end it is renewed and most frequently is the cause of death. Whoever saw a child with tubercular disease of the lungs recover to die of old age? or whoever saw the young of either sex, suffer from unmistakable tubercular disease with softening, or suppuration, yet recover to die in advanced life, of other disease?

It seems to me, that when we admit the tubercular nature of posterior curvature of the spine, we also admit its incurability and its almost certain progress to a fatal termination; such, however, is not the clinical history of spinal curvature. While its probable tubercular character has been universally admitted, the fact of frequent recovery has been faithfully observed. It is not true that spinal curvature progresses to a fatal termination, as a rule, exaggerated cases in very feeble, poorly nourished, half-made-up children prove fatal, or produce a condition of general paralysis worse than death; but the great majority of patients suffering from spinal curve of all forms recover in greater or less degree; often live useful and happy lives and die in old age or advanced life of other disease. This simple clinical fact, is to my mind conclusive evidence that generally it is not of tubercular origin and that if ever caused by tubercular disease, it can be only in rare and exceptional cases.

The vertebral bodies are like the cancellated structures in general, highly vascular, and susceptible to inflammation. This process in the bones, and its pathological results are well understood. There is not a single symptom or change connected with so-called Pott's disease, that could not be brought in harmony and fully explaned by supposing the disease to arise from inflammation. Its process and products produce all the symptoms and changes observed in the disease, and it cannot be necessary to suppose tuberculosis, or other hereditary or acquired dyscrasia to satisfactorily account for the causes, progress and termination of spinal curve. One important question yet remained to be settled, in the pathology of spinal curvature, viz.: whether at all, and to what extent the inter-vertebral cartilages partake in the establishment of the disease. The shape of these cartilages greatly influences and effects the natural curves of the spine in health; if this is so, it is natural to suppose that their integrity and form would greatly influence it in disease. Pathological anatomy has removed all doubt; caries of the vertebræ extend invariably to the fibro-cartilagenous structures, though the change in them does not appear to be so rapid as in the bones. The question, undecided perhaps then is, whether the fibro-cartilages may be the seat of primary affections? These cartilages are possessed of a high degree of vitality judging from their vascular supply. The elacticity and flexibility of the spine depends upon the cartilages, and it seems highly probable that undue strain or injury would lead to structural changes, and make them the seat of primary disease.

Having thus rapidly passed in review some of the pathological conditions which underlie posterior curvature of the spine, we may take into consideration the question, whether they are, such as admit of total or partial restoration to the natural form and functions. Good pathologists will many of them say, no! spinal doctors will say, yes! One will point you to their pathological observations and specimens; the other show you diagrams representing cases before and after treatment, fearful curves *before* and perfectly cured after,—cured by a patented *pair* of *stays*, or a spinal supporter, and done, too, in a remarkably stort time. This will be supported by the corroborative testimony of some very eminent physicians who observed the case and can speak from actual observation. But it is unnecessary for me to speak of all this *clap trap* of pretention before an audience of your intelligence and education. We shall therefore enter upon the pathological changes observed in this disease and the influences they exert. From this basis alone, can be deduced a rational answer. The case before you has proved fatal from long continued ulcerative disease of the vertebræ and inter-vertebral cartilages, culminating in psoas abcess and termininating in death by extensive suppurative inflammation of the peritoneum and general abdominal viscera, which are well shown by the autopsy; but no indications of tuberculosis can be seen in the lungs, or elsewhere.

